# Detection of African Swine Fever Virus in *Ornithodoros* Tick Species Associated with Indigenous and Extralimital Warthog Populations in South Africa

**DOI:** 10.3390/v14081617

**Published:** 2022-07-26

**Authors:** Anthony F. Craig, Mathilde L. Schade-Weskott, Thapelo Rametse, Livio Heath, Gideon J. P. Kriel, Lin-Mari de Klerk-Lorist, Louis van Schalkwyk, Jessie D. Trujillo, Jan E. Crafford, Juergen A. Richt, Robert Swanepoel

**Affiliations:** 1 Vectors and Vector-Borne Diseases Research Programme, Department of Veterinary Tropical Diseases, Faculty of Veterinary Science, University of Pretoria, Pretoria 0110, South Africa; af.craig83@gmail.com (A.F.C.); matschade@gmail.com (M.L.S.-W.); lvs@vodamail.co.za (L.v.S.); jannie.crafford@up.ac.za (J.E.C.); bob.swanepoel@up.ac.za (R.S.); 2 Agricultural Research Council-Onderstepoort Veterinary Research Transboundary Animal Diseases Laboratory, Onderstepoort, Pretoria 0110, South Africa; rametset@arc.agric.za (T.R.); heathl@arc.agric.za (L.H.); 3 Provincial Veterinary Services, Department of Agriculture, Land Reform and Rural Development, Kimberley 8300, South Africa; dkriel@ncpg.gov.za; 4 Office of the State Veterinarian, Department of Agriculture, Land Reform and Rural Development, Kruger National Park, P.O. Box 12, Skukuza 1350, South Africa; linmariedk@dalrrd.gov.za; 5 Department of Migration, Max Planck Institute of Animal Behavior, Am Obstberg 1, D-78315 Radolfzell, Germany; 6 Diagnostic Medicine/Pathobiology, Center of Excellence for Emerging and Zoonotic Animal Diseases (CEEZAD), College of Veterinary Medicine, Kansas State University, Manhattan, KS 66506, USA; jdtrujillo@vet.k-state.edu

**Keywords:** African swine fever virus (ASFV), sylvatic circulation, *Ornithodoros* ticks, South Africa

## Abstract

We investigated the possibility that sylvatic circulation of African swine fever virus (ASFV) in warthogs and *Ornithodoros* ticks had extended beyond the historically affected northern part of South Africa that was declared a controlled area in 1935 to prevent the spread of infection to the rest of the country. We recently reported finding antibody to the virus in extralimital warthogs in the south of the country, and now describe the detection of infected ticks outside the controlled area. A total of 5078 ticks was collected at 45 locations in 7/9 provinces during 2019–2021 and assayed as 711 pools for virus content by qPCR, while 221 pools were also analysed for tick phylogenetics. Viral nucleic acid was detected in 50 tick pools representing all four members of the *Ornithodoros (Ornithodoros) moubata* complex known to occur in South Africa: *O. (O.) waterbergensis* and *O. (O.) phacochoerus* species yielded ASFV genotypes XX, XXI, XXII at 4 locations and *O. (O.) moubata* yielded ASFV genotype I at two locations inside the controlled area. Outside the controlled area, *O. (O.) moubata* and *O. (O.) compactus* ticks yielded ASFV genotype I at 7 locations, while genotype III ASFV was identified in *O. (O.) compactus* ticks at a single location. Two of the three species of the *O. (O.) savignyi* complex ticks known to be present in the country, *O. (O.) kalahariensis* and *O. (O.) noorsveldensis*, were collected at single locations and found negative for virus. The only member of the *Pavlovskyella* subgenus of *Ornithodoros* ticks known to occur in South Africa, *O. (P.) zumpti*, was collected from warthog burrows for the first time, in Addo National Park in the Eastern Cape Province where ASFV had never been recorded, and it tested negative for the viral nucleic acid. While it is confirmed that there is sylvatic circulation of ASFV outside the controlled area in South Africa, there is a need for more extensive surveillance and for vector competence studies with various species of *Ornithodoros* ticks.

## 1. Introduction

African swine fever virus (ASFV) causes a contagious and lethal disease of domestic pigs. In the savannah areas of eastern and southern Africa, the virus is maintained in sylvatic circulation between warthogs that develop benign viraemic infection and eyeless ticks of the *Ornithodoros (Ornithodoros) moubata* complex that live in warthog burrows [1,2,3]. In South Africa, the disease in domestic pigs was first recognized in the north of the country, where the Common warthog (*Phacochoerus africanus*) was prevalent [4]. Consequently, a controlled area was declared in the north in 1935, and regulations were implemented to prevent the transfer of infected suids or products to the rest of the country [5].

The regulations initially proved effective. However, from 2016 there were outbreaks of the disease in domestic pigs in the south that could not be linked to the recent transfer of infected animals or materials from the controlled area. The genotype of ASFV involved, genotype I, had been isolated decades earlier from outbreaks of disease in pigs, and from ticks in South Africa. The same genotype had long been associated with the disease in domestic pigs in countries of western Africa from where it is believed to have been accidentally introduced into Europe in 1957 and 1960 [6,7,8,9,10,11,12,13,14]. In South Africa, there has been widespread translocation of warthogs since 1963 to game farms and nature reserves in the south of the country, originally from a source considered to be free of ticks and virus. In 2008–2012, *Ornithodoros* ticks were found in warthog burrows on farms approximately 20 km south of the controlled area, including ASFV-infected ticks in one instance [15,16,17,18,19].

**Figure 1:**
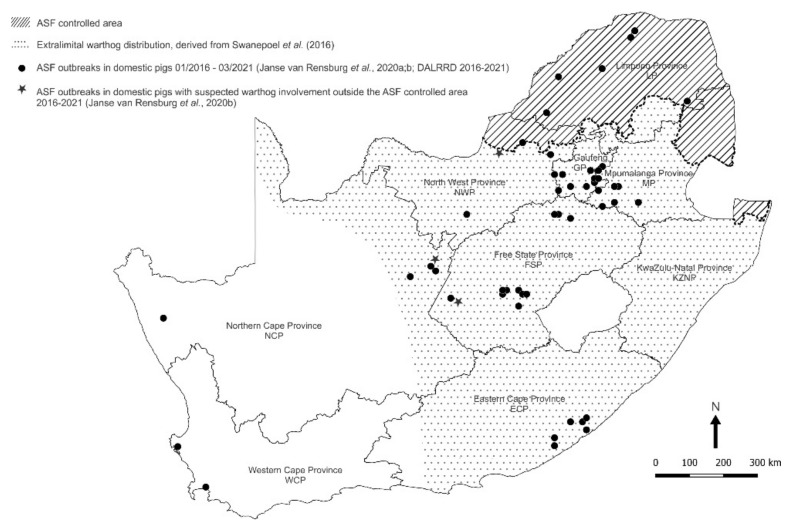
Spatial distribution of outbreaks of African swine fever in domestic pigs in relation to the controlled area and the extralimital distribution of warthogs in South Africa, 2016–2021. Approximate coordinates (correct to 0.1 degree) were derived from references cited in the text. The South African Protection of Personal Information Act 4 of 2013 precludes divulging names and accurate coordinates of private property.

Although the most recent outbreaks of the disease outside the controlled area were of undetermined origin, there were indications that contact with warthogs was involved in at least three instances in the North West, Northern Cape and Free State Provinces (Figure 1) [6,7,8,9,11,12]. Hence, we sought evidence of the occurrence of sylvatic circulation of ASFV outside the controlled area and found antibody to the virus in opportunistically acquired serum samples from extralimital warthogs [20]. We now report the detection of ASFV nucleic acid in *Ornithodoros* ticks collected from warthog burrows both within and beyond the controlled area in South Africa.

The taxonomy of the Afrotropical *Ornithodoros* ticks was recently revised with the description of new species [21], and the present observations incidentally extend the information available on the distribution ranges, host associations and potential roles of certain tick species as vectors of ASFV. Moreover, tests for antibody to ASFV were performed on a limited number of blood and serum samples from tortoises and hyaenas to determine whether they are exposed to infection as alternative hosts for *Ornithodoros* ticks.

## 2. Materials and Methods

### 2.1. Study Sites and Collection of Ticks

*Ornithodoros* ticks were collected in the same three national parks where warthog serum samples were obtained to test for antibody to ASFV [20]. The Greater Kruger National Park (GKNP) inside the controlled area was sampled as representative of an environment where circulation of ASFV in ticks and warthogs is endemic, while Addo Elephant National Park (Addo ENP) was included in the study for contrast since ASF had never been recorded in the Eastern Cape Province at the time of sampling in 2019, although there were outbreaks of the disease about 250 km east of the national park in the following year. Specific evidence of sylvatic circulation of ASFV was sought in Mokala National Park (Mokala NP) because contact with warthogs had been reported in outbreaks of the disease in domestic pigs in Northern Cape Province and adjacent western Free State Province (Figure 1). In addition, ticks were collected from private farms and nature reserves in seven provinces, either inside the controlled area or in the vicinity of past outbreaks of ASF in domestic pigs outside the controlled area (Table 1).

The ticks were collected during 2019–2021 and each property was designated as a separate location, except for the large GKNP where multiple locations were sampled. Within locations, tick collection sites were defined as single warthog burrows or clusters of burrows sometimes overtly inter-leading but separated from other sites on the same property by distances of ≥1 km. Assuming a minimal ASFV infection rate of 1% the intention was to collect at least 300 ticks per location to confirm the presence or absence of virus [22], but this number could not always be attained. Coordinates of collection sites were recorded according to the quarter-degree grid cell (QDGC) system [23].

Ticks of the eyeless *O. (O.) moubata* complex were collected with entomological forceps from sand scraped from the roofs, walls and floors of warthog burrows with a long-handled shovel, or from sand taken from culverts utilized by warthogs [24]. Members of the eyed *O. (O.) savignyi* complex that live in sand at animal resting sites instead of burrows, were reportedly present on four of the properties visited and attempts were made to collect them at sites indicated to be infested. Ticks were placed alive in labelled screw-top 40 mL plastic sample containers with loose filter paper liners and transported under veterinary permit in prescribed secondary and tertiary biosecurity packaging to the laboratory for examination under animal biosafety level 3 conditions. The ticks were held at laboratory temperature (22 °C) for 3 weeks prior to processing to reduce the detectability of residual virus that may have been engorged in viraemic blood meals [25].

Ticks were identified morphologically and a few specimens from each collection location were preserved in 70% ethanol as taxonomic vouchers for confirmation of identity by the Gertrud Theiler Tick Museum of the Agricultural Research Council-Onderstepoort Veterinary Research, Onderstepoort, South Africa. The remaining ticks were pooled according to sampling site, developmental instar and size into groups of 1–2 adults or 5–25 nymphs and stored at −80 °C until virus and phylogenetic assays were performed.

### 2.2. Nucleic Acid Extraction and Gene Amplification

Tick pools were homogenized using 1.4 mm zirconium oxide beads in centrifuge tubes containing phosphate-buffered saline, pH7.2 (PBS) to create 10% (*w/v*) suspensions. Automated nucleic acid extraction was performed with IndiMag Pathogen kits (Indical Bioscience, Leipzig, Germany) using slight modifications to the manufacturer’s instructions. Briefly, 200 µL of tick homogenate supernatant was added to 200 µL AL buffer (Qiagen), mixed well, and incubated at 70 °C for 10 min. 200 µL of the AL lysate was added to the IndiMag buffer for extraction. Each extraction included known ASFV positive controls.

Eluates were stored at −80 °C until tested for ASFV nucleic acid using the real-time quantitative PCR (qPCR) assay of Zsak et al. [26] with modifications [27]. Briefly, 5 µL DNA was amplified in 20 µL reactions using 20 pmol of the published primers and 7 pmol of probe in Perfecta Fastmix II (Quanta Biosciences, Beverly, MA, USA). Positive and no template controls (NTC) were included for each PCR run. Samples with Cq mean values ≤38 (selected as the cut-off value based on the analytical sensitivity limits of the qPCR assay) were considered positive.

To confirm the identification of ASFV detected in tick pools and classify the virus genotypes involved, nucleic acid from qPCR-positive pools was amplified with primers p72-U and p72-D and the cycling conditions of Bastos et al. [10]. Appropriately-sized bands (~478 bp) of amplification product were excised from agarose electrophoresis gels, purified, and subjected to Sanger nucleotide sequencing. The sequences were viewed, 399 base fragments of DNA were aligned using MEGA X software [28] and neighbour-joining phylogenetic analysis was performed with representative sequences of the 10 ASFV p72 genotypes confirmed to occur in South Africa plus one unassigned virus (L. Heath, unpublished) using MEGA X software [28,29].

To confirm the potential significance of intra-genotypic single nucleotide polymorphisms (SNPs) observed among p72 virus genotype I sequences, partial characterization of the central variable region (CVR) of the 9RL open reading frame of ASFV was performed on the relevant tick pool DNA extracts using the PCR primers and cycling conditions of Bastos et al. [30] followed by nucleotide sequencing of the products and deduction of amino acid sequences.

Phylogenetic characterization of ticks was based on partial sequencing of the mitochondrial 16S rRNA gene [31]. Appropriately sized bands (~313 bp) were excised from agarose electrophoresis gels, purified, and subjected to Sanger nucleotide sequencing. The sequences were viewed, 263 base fragments aligned, and neighbour-joining phylogenetic analysis performed with representative species sequences from GenBank using MEGA X software [28].

### 2.3. Isolation of Virus

In attempts to isolate virus from ASFV qPCR-positive tick pools, aliquots of clarified supernatant fluid from the original 10% suspensions were inoculated at 10-fold dilutions into duplicate wells of primary pig bone marrow macrophage cultures in 96 well microplates and examined for hemadsorption (HAD) and cytopathic effect (CPE) at 48 and 72 h incubation [32]. Negative samples were scheduled to be sub-cultured twice, and isolation of virus in HAD- or CPE-positive samples confirmed by performing qPCR [27] on culture extracts.

### 2.4. Identification of Tick Blood Meal Donor Species

In attempts to identify blood meal donors, partial cytochrome b sequences of mammalian mitochondrial DNA were determined from selected suspensions of pools of engorged ticks [33] and compared by BLASTx search with sequences in GenBank (NCBI).

### 2.5. ASFV Antibody Tests on Alternative Vertebrate Hosts of Ornithodoros Ticks

Dried blood samples were collected on Nobuto cellulose strips (NCS) (Advantec, Tokyo, Japan) from 5 leopard tortoises (*Stigmochelys pardalis*) killed in road accidents in the Kimberley area of Northern Cape Province and in the southern GKNP in 2020–2021. Serum samples from 97 spotted hyaenas (*Crocuta crocuta*) obtained from the GKNP came from animals translocated internally or culled for managerial purposes in the south of the national park from 1997 to 2018 and had been stored at −80 °C. Tests for antibody to ASFV were performed on the blood and serum samples with INgezim PPA Compac R. 11.PPA.K3 blocking enzyme-linked immunosorbent assay (ELISA) kits (Eurofins Technologies Ingenasa, Madrid, Spain) as described previously [20].

## 3. Results

### 3.1. Collection and Identification of Ornithodoros Ticks

In total, 5078 ticks were collected at 82 sites in 45 locations in 7/9 provinces of South Africa during 2019–2021. An additional 23 sites in 14 locations failed to yield ticks, including a nature reserve in Mpumalanga Province bordering Gauteng Province, plus 7 small contiguous farms in north-eastern GP where acaricides were used liberally, and 6 farms in western Gauteng Province where warthogs were rarely seen, and the burrows appeared to be occupied by porcupines (Figure 1; Table 1). Ticks were identified morphologically, and a subset of 436 ticks was preserved as taxonomic vouchers. The remaining 4642 ticks were assayed in 711 pools for virus content.

Partial mitochondrial 16S rRNA gene sequences were determined for 221 tick pools, including pools that tested positive for ASFV nucleic acid plus at least one pool per collection location and all pools from some locations, except for location LP02 where inadequate DNA remained available (GenBank accession numbers MZ411417-MZ411419, OK136965-OK137179, OK323967, OK323968 and OL870945) (Table 1).

**Figure 2:**
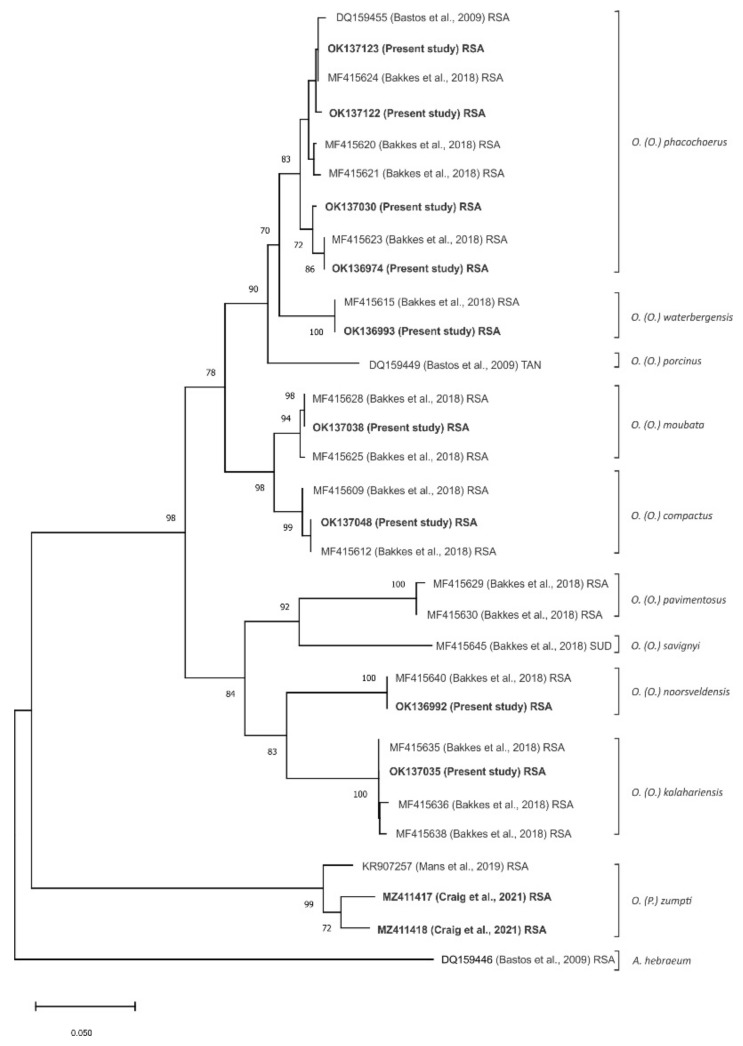
Neighbour-joining tree based on partial mitochondrial 16S rRNA gene sequence (263 nt) depicting phylogenetic relationships between 11 unique sequences generated from African *Ornithodoros (Ornithodoros)* ticks in the present study (GenBank accession numbers in bold) and 19 representative unique *Ornithodoros* species sequences from Genbank, mainly from South Africa (RSA) but including *O. (O.) porcinus* Walton, 1962 from Tanzania (TAN) and *O. (O.) savignyi* (Audouin, 1826) from Sudan (SUD), plus the ixodid tick *Amblyomma hebraeum* as an outlier. Percentage bootstrap support values were derived following 10,000 replications. Evolutionary analyses were conducted in MEGA X.

All 221 partial mitochondrial 16S rRNA tick gene sequences were aligned and subjected to neighbour-joining phylogenetic analysis [28] with 74 corresponding gene sequences obtained from GenBank for Afrotropical *Ornithodoros* ticks, mainly from South Africa but including *O. (O.) porcinus* Walton, 1962 from Tanzania and *O. (O.) savignyi* (Audouin, 1826) from Sudan, plus the ixodid tick *Amblyomma hebraeum* as an outlier. The 221 data sets from the present study resolved into 11 unique sequences while the 74 data sets from GenBank represented 19 unique *Ornithodoros* sequences, corresponding to a collated total of 23 unique sequences clustering as 10 currently recognized species of *Ornithodoros* ticks [21] (data not shown). For sake of clarity, the analysis was repeated using only representative unique sequences from the present study and from the GenBank data, resulting in the generation of a dendrogram showing the same topology and phylogenetic relationships as the full range of data sets (Figure 2).

The ticks collected in the present study were found to include all four species of the *O (O.) moubata* complex known to occur in South Africa: *O. (O.) moubata* (Murray) (1877) sensu Walton, 1962, *O. (O.) waterbergensis* Bakkes et al., 2018, *O. (O.) phacochoerus* Bakkes et al., 2018 and *O. (O.) compactus* Walton, 1962, each within a distinct distribution range (Table 1). Furthermore, two of the three species of the *O. (O.) savignyi* complex ticks known to be present in the country, *O. (O.) kalahariensis* Bakkes et al., 2018 and *O. (O.) noorsveldensis* Bakkes et al., 2018, were found at single locations in Northern Cape Province and Eastern Cape Province. In addition, the only member of the subgenus *Pavlovskyella* yet recorded in South Africa, *O. (P.) zumpti* Heisch and Guggisberg, 1953, was collected from warthog burrows and a yellow mongoose (*Cynictis penicillata*) burrow in Addo NP in Eastern Cape Province (Table 1).

### 3.2. Detection and p72 Phylogeny of ASFV Nucleic Acid in Ticks

**Figure 3:**
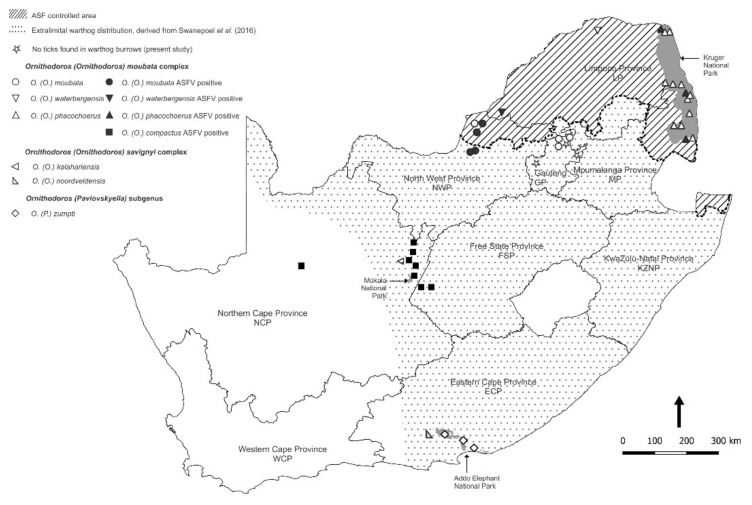
Spatial distribution of locations where different *Ornithodoros* tick species were collected during the present study. Locations where African swine fever virus nucleic acid was detected in ticks (closed symbols) are shown in relation to locations where viral nucleic acid was not found in ticks (open symbols).

Of the 711 pools of ticks screened by qPCR for ASFV nucleic acid, 50 pools tested positive with a Cq mean value of 26.00 (range 20.23–36.11), including members of all four of the *O (O.) moubata* complex sampled. A further 10 pools of ticks produced doubtful reactions in the qPCR and failed to react in the p72 PCR or could not be sequenced, and the findings were discarded since the presence of the virus was already confirmed in other tick pools at the same collection sites. As expected, the few *O. (O.) kalahariensis* and *O. (O.) noorsveldensis* ticks of the *O. (O.) savignyi* complex that do not live in burrows failed to yield ASFV nucleic acid, as did the *O. (P.) zumpti* ticks from Eastern Cape Province where the presence of the virus had never been recorded at the time that the ticks were collected (Table 1). The locations where ASFV nucleic acid was detected in ticks collected during the present study are shown in Figure 3 in relation to locations where ASFV was not found in ticks.

**Figure 4:**
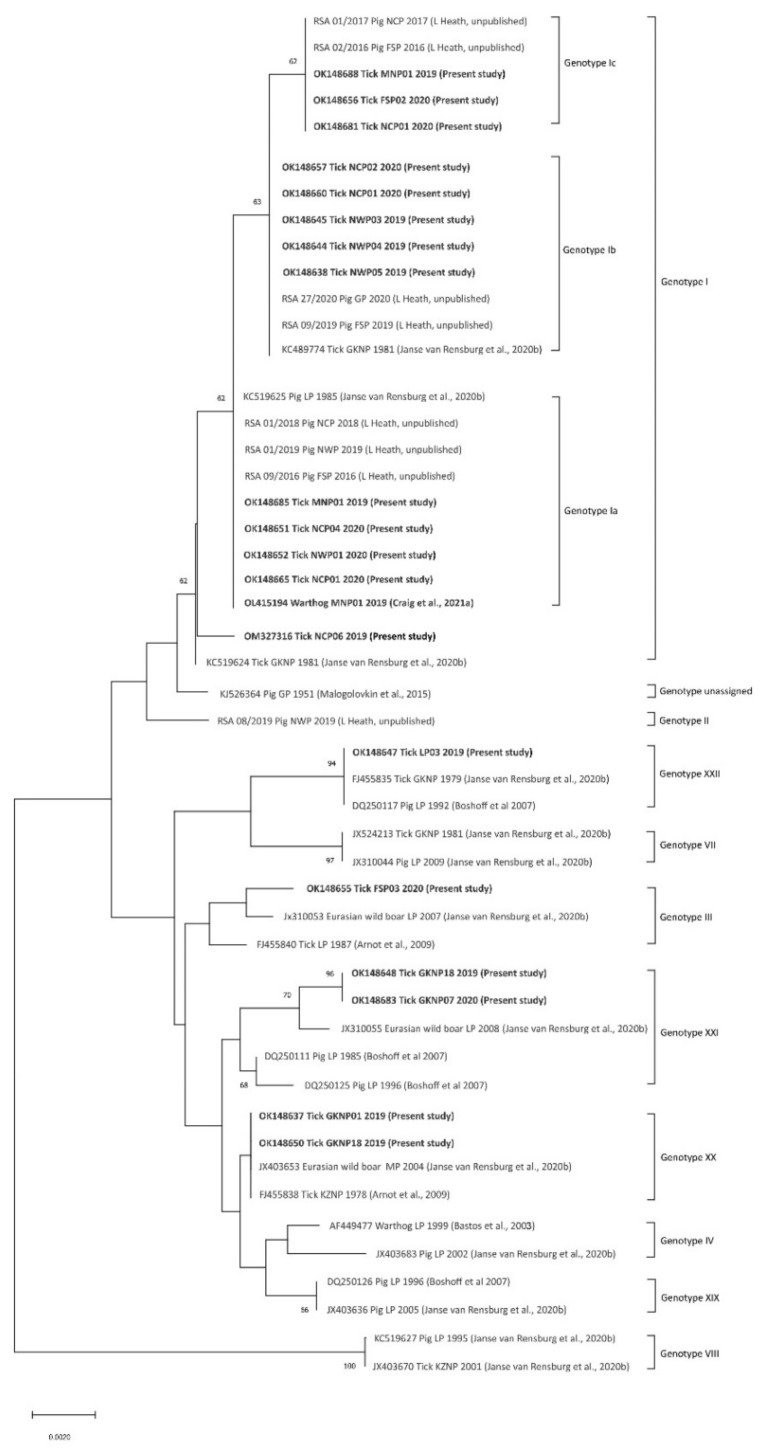
Neighbour-joining tree based on partial C-terminal p72 gene sequence (399 nt) of African swine fever virus depicting phylogenetic relationships between 20 representative sequences detected in African *Ornithodoros (Ornithodoros)* ticks in the present study (GenBank accession numbers in bold) and 29 representative sequences of the genotypes known to occur in South Africa. Only one unique sequence detected per tick species per collection site in the present study is included in the analysis and these comprise 5 genotypes of virus. Percentage bootstrap support values were derived following 10,000 replications. Evolutionary analyses were conducted in MEGA X.

All 50 of the qPCR-positive tick pools also tested positive in the p72 PCR and phylogenetic analysis revealed that the ASFV nucleic acid detected in the ticks represented five p72 genotypes of the virus (Figure 4; Table 1), with SNPs that distinguished 4 variants of genotype I supported by marked size differences in the CVR compatible with subtypes recorded in recent outbreaks of the disease in domestic pigs outside the controlled area [13]; see *Discussion* below. Only 20 sequences from the present study were included in the analysis, comprising one unique partial p72 sequence per tick species per collection site plus one sequence (GenBank accession number OL415194) from a warthog [20], along with 29 representative sequences of the genotypes known to occur in South Africa including isolate Spencer that has not been assigned to a p72 genotype; see *Discussion* below. The inclusion of a type II isolate (RSA 08/2019, Figure 4) (L. Heath, unpublished) in the analysis incidentally raises the number of known genotypes confirmed to be present in South Africa to 10, but an update on ASFV isolations from recent outbreaks of the disease will be presented separately. In the present study, ticks of the *O. (O.) waterbergensis* and *O. (O.) phacochoerus* species yielded ASFV genotypes XX, XXI and XXII at 4 locations and *O. (O.) moubata* yielded 2 subtypes of ASFV genotype I at two locations inside the controlled area. Outside the controlled area, *O. (O.) moubata* and *O. (O.) compactus* ticks yielded 4 subtypes of ASFV genotype I variously at 7 locations with 3 pools of *O. (O.) compactus* ticks yielding two subtypes, Ia and Ib, of genotype I at location NCP01. In addition, genotype III ASFV was identified in *O. (O.) compactus* ticks at a single location outside the controlled area in western Free State Province (GenBank accession numbers OK148637-OK148688 and OM135580) (Table 1). Two separate GenBank submissions were made for each of three tick pools that yielded subtypes Ia and Ib of genotype I ASFV (Table 1). The detection of genotype III virus outside the controlled area was confirmed by re-extraction of an aliquot of the original tick suspension and repeating the partial characterization of the p72 gene.

**Figure 5:**
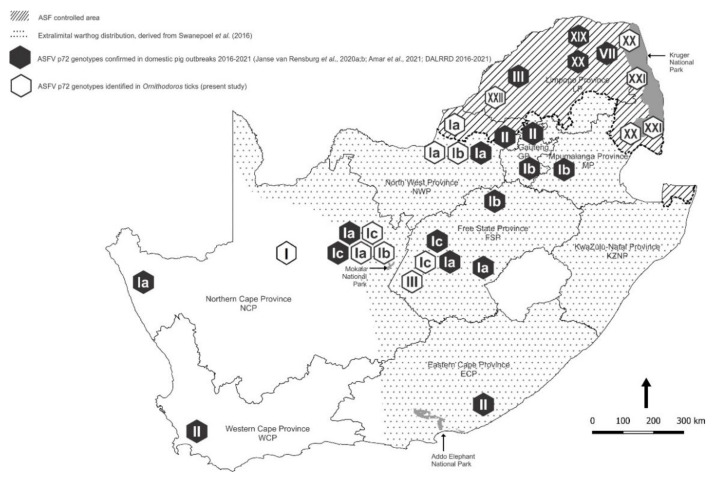
Spatial distribution patterns of the African swine fever virus genotypes identified in ticks during the present study (open symbols) summarized on a regional basis in relation to virus genotypes identified in outbreaks of African swine fever in domestic pigs (closed symbols) from 2016–2021.

**Figure 6:**
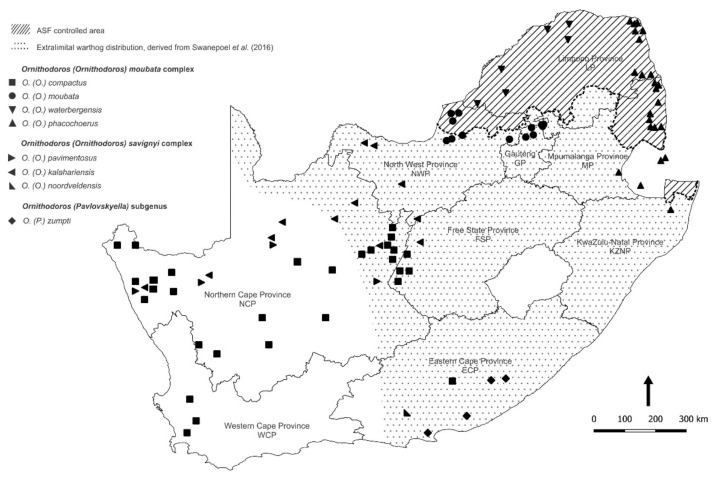
Updated distribution ranges of the currently recognized species of *Ornithodoros* ticks in South Africa based on locations where ticks were collected during the present study together with locations where ticks with cognate partial mitochondrial 16S RNA gene sequences or morphological identity were previously reported by sources cited in the text.

The distribution patterns of the ASFV genotypes identified in ticks during the present study are summarized on a regional basis in Figure 5 in relation to virus genotypes identified in outbreaks of ASF in domestic pigs during 2016–2021 [6,7,8,9,12,12,34]. The extant information on the distribution ranges of the currently recognized species of *Ornithodoros* ticks in South Africa is collated in Figure 6 by plotting the locations where ticks were collected during the present study in relation to locations where ticks with cognate partial mitochondrial 16S RNA gene sequences or morphological identity were previously reported [21,31,35,36,37,38].

### 3.3. Isolation of Virus

In an initial attempt to isolate virus from ASFV qPCR-positive tick pools, 6/18 samples produced positive HAD results on first pass in cell cultures, but further investigations had to be deferred since the preparation of macrophage cultures was suspended during the COVID-19 pandemic. From qPCR performed on laboratory stock ASFV that is routinely titrated as a control with each batch of samples cultured for virus isolation, it was extrapolated that tick suspensions that had qPCR Cq values of ≤22 probably had infective titres ≥10^6.0^ HAD_50_/mL. This applied to at least one tick pool from each of the *O. (O.) phacochoerus*, *moubata* and *compactus* samples tested, while the single *O. (O.) waterbergensis* qPCR-positive tick suspension had an extrapolated titre of ≥10^4.0^ HAD_50_/mL.

### 3.4. Molecular Identification of Blood Meal Hosts

Determination of partial cytochrome b sequences of mammalian mitochondrial DNA [33] was attempted on suspensions of 64 pools of partially engorged ticks, including pools that yielded ASFV nucleic acid, but only one pool of *O. (O.) phacochoerus* ticks from collection location GKNP01 and one pool of *O. (P.) zumpti* ticks from location AENP02 produced sequences, and a BLASTx search of GenBank (NCBI) confirmed that warthogs served as blood meal donors for ticks in both pools.

### 3.5. ASFV Antibody Tests on Hyaena and Tortoise Sera

Dried blood samples from 2 leopard tortoises (*Stigmochelys pardalis*) collected in southern GKNP and 3 collected in the Kimberley area of Northern Cape Province plus 97 serum samples from spotted hyaenas in the GKNP all tested negative for antibody to ASFV.

## 4. Discussion

The repeated detection of viral nucleic acid in *Ornithodoros* ticks and the high prevalence of antibody observed in the sera of extralimital warthogs [20] are suggestive of extensive sylvatic circulation of ASFV beyond the controlled area in South Africa that warrants further investigation. However, the full implications of the present findings are best assessed in relation to the extant information on sylvatic ASFV.

There are over 100 known species of *Ornithodoros* ticks worldwide; the exact number varies with periodic discoveries and taxonomic revisions [21,38,39,40,41]. The resistance to desiccation conferred by the wax and cement layers of their cuticles facilitates their occurrence in the warmer and more arid regions of all continents [42,43,44]. The ticks are nidicolous, living in close association with their hosts. A distinction is made between endophilous nidicoles such as *O. moubata* sensu lato that occur within host habitations including animal burrows, and harbourage nidicoles such as *O. savignyi* s.l. that live in the environs of their hosts, for example in the sand at animal resting sites, although differences in behaviour are not always clear-cut [43,45].

Several African and exotic *Ornithodoros* species have been found capable of sustaining replication and transmission of ASFV experimentally, but vector competence appears to be regulated by multiple tick species- and virus strain-specific factors [46,47,48,49,50,51,52,53,54,55,56,57,58,59,60,61,62,63,64,65,66,67,68,69,70,71,72,73]. Hence, it is important that both ticks and viruses should be identified accurately in assessing and recording vector potential.

Endophilous Afrotropical *Ornithodoros* species adapt readily to living in the cracks and crevices of rustic human dwellings and livestock shelters, and this influenced the manner in which the taxonomy of the ticks evolved (Table 2). The type species of the genus *Ornithodoros, O. savignyi* (Audouin, 1827), has eyes and was originally described as *Argas savignyi* Audouin, 1827 from specimens collected from sand in Egypt (Table 2) [74]. The eyeless *O. moubata* (Murray) (1877), sensu Walton, 1962, was described as *Argas moubata* Murray, 1877 from a specimen collected in Angola where the presence of the ticks in human dwellings had been noted many years earlier [75]. Both species were subsequently transferred to the genus *Ornithodoros* which was erected by Koch [76] and is sometimes rendered incorrectly as *Ornithodorus* [77]. There was also description of an eyeless *O. savignyi* var. *caeca* Neumann (1901), occasionally reported as *caecus* or *caecum*, but this was soon synonymized with *O. moubata* [78] and is omitted from Table 2. An additional eyed species, *O. (O.) pavimentosus* Neumann, 1901, was described from a single specimen from Namibia but later synonymized with *O. savignyi* [79], and subsequently resurrected with re-description based on neotype specimens from Northern Cape Province, South Africa [21].

By the late nineteenth and early twentieth century, *Ornithodoros* ticks were widely known to inhabitants of Africa as infesting dwellings and inflicting painful bites while people slept at night, sometimes transmitting a potentially fatal illness that proved to be tick-borne relapsing fever (TBRF) caused by borrelias [75,80,81,82,83,84]. Investigators of TBRF routinely distinguished between *O. savignyi* s.l. and *O. moubata* s.l. based on the presence or absence of eyes. Although it was known that livestock sharing human dwellings at night were also bitten, *O. moubata* s.l. was considered to be primarily a parasite of humans, prevalent from Eritrea in the north to as far south as Graaff-Reinet in South Africa, and including present-day Ethiopia, South Sudan, Somalia, Uganda, Kenya, Tanzania, Malawi, Mozambique, Zambia, Zimbabwe, Namibia, Botswana, Angola and Madagascar, with incursion westwards into the Democratic Republic of the Congo (DRC), Congo Republic and Cameroon along trade routes [75,85,86,87,88,89,90].

Transmission of ASFV by *O. (P.) erraticus* ticks was discovered in 1963 in Spain following the accidental introduction of the virus into Europe [91] and prior to this event veterinary interest in *Ornithodoros* ticks was relatively limited in Africa. Nevertheless, on various occasions from 1907 to 1943 the presence of *O. moubata* s.l. ticks was recorded in pigsties in Angola, DRC, Zimbabwe, South Africa and Malawi, warthog burrows in Zambia and DRC, on a warthog shot in Uganda, in association with poultry and other domestic animals in South Africa and, notably, was collected as *Argas moubata* from 44 tortoises in the environs of Niekerkshoop in Northern Cape Province, South Africa [92,93,94,95,96,97,98,99,100,101]. The investigators of TBRF were prompted to explore the possibility that *O. moubata* s.l. had sylvatic origins, possibly in association with large burrowing animals such as warthogs and porcupines [90,102,103]. Evidence emerged that *O. moubata* s.l. had also been found on elephant, lion, hyaena, Ground pangolin (*Smutsia temminckii*), antbear (*Orycteropus afer)* and domestic cattle in addition to warthogs and porcupines in Angola and Mozambique [104,105,106]. Walton [90,103,105,106,107,108] collected *O. moubata* s.l. ticks from different locations and domestic or wild habitats and compared the duration of their life cycles and longevity under laboratory conditions to identify a range of ‘biological forms’ that implied the taxon was not monotypic. Limited observations on the viability and teratology of hybrids tended to support the distinctness of the biological forms.

Walton [105,109] noted that the designation *O. moubata* (Murray) 1877 was a nomen dubium since the description was inadequate and the type specimen was lost, and he replaced it with four species and a subspecies derived from the biological forms that he had identified (Table 2). He used ticks of the ‘Groot Marico strain’ derived from human dwellings on a property (location NWP02, Table 1) north of a village of that name in South Africa as neotypes for re-description of *O. moubata* since they were found to be ‘identical biologically’ to ticks from Angola and Namibia. The re-described *O. moubata* was regarded as being associated particularly with human dwellings in southern Africa but was stated to range in distribution north-eastwards to Tanzania, while the possibility and extent of its occurrence in wild habitats was considered speculative [106]. A new species, *O. porcinus,* was erected for ticks associated with warthog burrows in East Africa, but its distribution was postulated to extend to southern Africa, while a new subspecies, *O. porcinus domesticus,* was described as highly prevalent in human dwellings in East Africa [105,106]. The two remaining new species of Walton were considered to be limited to wild habitats; *O. apertus* associated with porcupine (probably *Hystrix cristata*) burrows in Kenya and *O. compactus* found on tortoises in the south-western region of South Africa, while *O. savignyi* was retained as valid [105,106]. The utility of Walton’s qualitative morphological descriptions and the validity of his biological observations were questioned by van der Merwe [110] who proceeded to merge the subspecies *O. p. domesticus* with *O. p. porcinus*, and re-assigned the taxa *moubata*, *porcinus* and *apertus* as subspecies of *O. moubata*, but retained *O. compactus* as a distinct species. However, Walton [111] re-affirmed his classification of the *O. moubata* complex and added a further subspecies, *O. p. avivora*, that parasitized domestic chickens along the coast of East Africa (Table 2).

Public health measures and the use of increasingly effective antibiotics and acaricides ultimately led to control of TBRF although it remains a threat [75,105,106,112,113,114], while interest in ASFV intensified. Black and Piesman [115] applied phylogenetic analysis based on partial sequencing of the mitochondrial 16S rRNA gene to ixodid and argasid ticks, and the incorporation of this approach into ASFV investigations revealed the existence of three geographically discrete lineages of *O. porcinus* s.l. but provided no support for recognition of *O. p. porcinus* and *O. p. domesticus* subspecies [31,36,37]. Bakkes et al. [21] duly undertook taxonomic revision of the Afrotropical *Ornithodoros (Ornithodoros)* subgenus and since the ticks are lacking in highly variable physical features useful for delimitation of species, they resorted to morphometric analysis of the profile of dorsal protuberances on the tarsal segment of the first leg to distinguish species that were corroborated by the lineages they generated in phylogenetic analysis. The *O. moubata* species of Walton [105] was retained as a parasite of warthogs, other burrowing animals, livestock and humans in north-western South Africa and adjacent countries, but historic records from East Africa were regarded as unconfirmed. Similarly, *O. porcinus* was retained as parasitizing warthogs, other wildlife, humans and livestock in East Africa. A new species, *O. phacochoerus,* was described as being associated with warthogs, livestock and humans in eastern South Africa and contiguous countries, while *O. waterbergensis* was erected as a new species with a similar host range in north-western South Africa. The *O. apertus* species of Walton was retained as being associated with porcupines in East Africa, and *O. compactus* as occurring on tortoises in south-western South Africa. Bakkes et al. [21] postulated that *O. savignyi* sensu stricto is restricted to northern Africa and the Near East, and they replaced it in the south-west of the African continent with a new species *O. kalahariensis* described as partially sympatric with *O. pavimentosus*, a species that they resurrected from synonymy. Finally, they erected *O. noorsveldensis* as a new eyed species known from a single locality in Eastern Cape Province, South Africa (location ECP01, Table 2). Since members of the *O. savignyi* complex are not associated with burrows it has been surmised that they are not involved in sylvatic circulation of ASFV, although *O. savignyi* s.s. was shown to be capable of transmitting the virus experimentally [24,50,116].

The identities of ticks collected during the present study are consistent with the classification of Bakkes et al. [21] and extend the known distribution ranges of their eyeless species in congruent manner, but we did not determine geographic limits of occurrence or areas of sympatry (Table 1; Figure 2 and Figure 6). Although morphometric analysis can be impractical for use in large-scale surveys, ASFV vector studies usually involve molecular assay of individual ticks for virus content that allows for convenient incorporation of tick phylogenetics [31]. Hence, the taxonomic classification of Bakkes et al. [21] should find general application, particularly since they anticipated that further studies could reveal additional novel species of ticks.

The distribution ranges of *Ornithodoros* ticks in South Africa as plotted in Figure 6 incorporate sites generated in the present study along with those given by Bakkes et al. [21] including records in their supplementary information, as well as records given by the same team in Mans et al. [38], and sites deduced by relating sequence data from past publications [31,36,37] to the current taxonomy. The sea bird parasite *Ornithodoros (Alectorobius) capensis* Neumann, 1901 was excluded as irrelevant to the present study although it would probably feed readily on any host species that impinged on its specialized habitats [43,45].

It is notable that *O. compactus* was described as a novel species by Walton [105] based on ‘*Argas moubata*’ specimens collected in 1932 from 44 tortoises of two species in Niekerkshoop, South Africa, as reported by Bedford [98], plus additional specimens from tortoises in Northern Cape Province, South Africa, as well as ticks collected from a tortoise in Hamburg Zoological Gardens and from the Reptile House in Regent’s Park Zoological Gardens, London. The same tick species was found in ‘tortoise burrows’ at two sites in Northern Cape Province, South Africa, (supplementary information) [21,38]. Moreover, 507 *O. compactus* ticks, including some adults, were reportedly found on 55 tortoises of seven species and subspecies, mainly in Western Cape and Northern Cape Provinces, South Africa, but also in adjacent Eastern Cape and Free State Provinces [35]. The ticks apparently lodge in the axial and perineal skin folds of tortoises [105]. Since all distribution records of *O. compactus* prior to the present study relate at least indirectly to tortoises, we have accepted the distribution records of Horak et al. [35] and incorporated them in Figure 6.

We not only found *O. compactus* to occur commonly in warthog burrows in Northern Cape Province and western Free State Province, South Africa, but detected a high prevalence of ASFV in these ticks, including virus nucleic acid in 19/51 pools tested from location NCP01 (Table 1). We were prompted to investigate whether tortoises might be involved in circulation of ASFV but managed to obtain only 5 dried blood samples from road kills that carried no *Ornithodoros* ticks and tested negative for ASFV nucleic acid and antibody. There are approximately 50 species of terrestrial tortoises in existence worldwide, with Africa being particularly rich in diversity. Some 14 species and subspecies of tortoises occur in the south-western region of the African continent with 12 of them being endemic, including threatened and endangered species subject to illegal international trade [117,118,119]. Even if tortoises prove to be refractory to ASFV infection, and despite their slow mobility, they could carry infected ticks long distances by air and other means of transport, which constitutes good reason to strengthen control of the illegal trade in endangered animals such as tortoises and pangolins. Furthermore, the observations on *O. compactus* reinforce the perception that *Ornithodoros* ticks are facultative with regard to blood meal donors and habitat so the naming of tick species for putative hosts could be misleading.

The hyaena sera were tested in parallel with the tortoise blood samples to check whether non-suids, which are potentially exposed to ASFV infection, as in *Ornithodoros* tick-infested burrows or culverts, produce detectable antibodies irrespective of their ability to sustain replication of the virus. Although previous investigators also failed to detect antibody to ASFV in non-suids, the numbers of samples that have been examined are relatively limited and the tests used in early studies were less sensitive than the blocking ELISA [120]. The poor results obtained in attempts to identify donors of blood meals probably relate to the fact that the test used is better suited to dipteran vectors, and improved techniques are available for use on ticks, while testing for antibody to *Ornithodoros* salivary antigens would have constituted a useful screening method to identify potential hosts of the ticks [121,122].

Although the high prevalence of antibody in warthogs [20] and the presence of the virus in ticks outside the controlled area in South Africa are consistent with sylvatic circulation of ASFV [3,24,116,123], definitive vector competence studies remain desirable, particularly for *O. (P.) zumpti* ticks, but lay beyond the scope of the present project. A short communication on finding *O. (P.) zumpti* ticks in warthog burrows was published separately to stimulate field investigations in the vicinity of ongoing outbreaks of ASFV infection in Eastern Cape Province [124]. Other ticks of the subgenus *Pavlovskyella,* known to be present in Africa, comprise members of the *O. (P.) erraticus* complex, including *O. (P.) marocanus* and *sonrai* that have distribution ranges extending from the Iberian Peninsula to the north African littoral and West Africa. The occurrence of ASFV nucleic acid was demonstrated in *O. (P.) sonrai* ticks collected in proximity to pigsties in Senegal, but the epidemiological significance of the finding was considered doubtful [125].

Viruses from the present study will be fully characterized as circumstances permit. Meanwhile, it is notable that SNPs observed among partial p72 sequences of genotype I isolates in South Africa were stable within recent series of outbreaks of disease in pigs connected by the spread of infection [13] and were supported by differences in product sizes of the CVR of the 9RL open reading frame of ASFV (L. Heath, unpublished). It is clear in retrospect that variants of genotype I had been isolated decades earlier from outbreaks of disease in pigs and from ticks, putatively *O. (O.) phacochoerus*, collected in GKNP in 1981 (Figure 4) [13,34]. Preliminary designation of some of the variants as subtypes Ia, b and c [13] should not be confused with the same designations applied to ASFV genotypes defined by p54 gene sequences [126], and the ambiguity will be addressed in a fuller description of the phenomenon. The present recovery of multiple subtypes of genotype I ASFV from ticks collected at single locations (Table 1), even from the same burrows, appears to be a novel observation.

Virus (GenBank accession number OM135580) identified in *O. compactus* ticks at location NCP06 (Table 1; Figure 3 and Figure 5), which lies beyond the western limit of the known distribution of warthogs as plotted in 2016 [18], may represent a further variant of genotype I, closest to a 1981 tick isolate from GKNP (Figure 4). Apparently, the presence of warthogs on property NCP06 relates to translocations made to a neighbouring property during the 1990s. Many unrecorded transfers of warthogs were made to private properties following the original translocations to nature reserves in the south of the country conducted by conservation officials during the 1960s and 1970s [17,127]. Interestingly, isolate Spencer that does not correspond to any existing p72 genotype and is represented in Figure 4 by GenBank accession number KJ526364 [128,129], was originally obtained in 1951 from an outbreak of the disease in southern Gauteng Province following the introduction of infection from Namibia [130,131]. Hence, its close relationship to recent Namibian isolates is understandable [132].

The detection of genotype III ASFV in ticks at a single location outside the controlled area (FSP03, Table 1) is difficult to explain. This is a small property in western Free State Province where few ticks were found in a single cluster of warthog burrows and there was no history of the disease in pigs kept in a fully enclosed building. Genotype III ASFV had previously been found in outbreaks of disease in pigs and in ticks at several locations only within the controlled area between 1993 and 2017 [34]. In contrast, the range of viruses detected in ticks collected within the controlled area in the present study (Table 1) is typical of the variety of genotypes of ASFV known to circulate in areas where sylvatic circulation of ASFV is endemic [14].

Subtype Ic of ASFV was identified in *O. (O.) compactus* ticks collected from burrows on property FSP02 (Figure 1 and Figure 3; Table 1) outside the controlled area in western Free State Province where the same subtype had been identified in 2016 in an outbreak of disease in free-ranging pigs that were suspected to have had contact with warthogs, including carcasses left lying in the fields. Subtypes Ia and Ib of ASFV were identified in *O. (O.) compactus* ticks collected from burrows on property NCP02 (Figure 1 and Figure 3; Table 1) where subtype Ia had caused an outbreak of disease in 2017 in penned pigs fed fresh entrails of a warthog shot on the same property. Although the infectivity of warthog offal for domestic pigs is disputed, low doses of the virus were shown to be infective for pigs by mouth [133] and transmission of the virus by the feeding of warthog entrails on property NCP02 tends to confirm this observation. For biosafety and legal reasons, only government veterinary officials are permitted entry to premises under quarantine following diagnosis of ASF outbreaks, so it was not possible to access a farm in North West Province only 10 km south of the controlled area where domestic pigs and Eurasian wild boars that escaped from their pens succumbed to the disease in 2019 after suspected contact with warthogs (Figure 1), but we detected ASFV nucleic acid in 7/39 pools of *O. (O.) moubata* s.s. ticks collected on two neighbouring properties; locations NWP04 and NWP05 (Figure 3; Table 1). The virus subtype detected, Ib, corresponds to that recovered in the disease outbreak. Since warthogs are not contagious for each other or domestic pigs [134,135], reference to transmission by contact with warthogs above is meant to imply consumption of infected warthog tissues or transmission through the intermediary of ticks. Thus, there is strong evidence that the virus in sylvatic circulation outside the controlled area has ignited infection in domestic pigs on occasion. However, no indication of the involvement of warthogs or ticks was obtained in the extensive series of outbreaks of disease that occurred in pigs in southern Gauteng Province and adjacent south-western Mpumalanga Province in 2016–2020 [12,13,136].

In 2018 there was an outbreak of disease associated with ASFV subtype Ia on a property in western Northern Cape Province (Figure 1 and Figure 5) in pigs shortly after they had been introduced from a farm in the Kimberley area that remained free of infection [6,7,8,9]. Accordingly, it was intended to extend the current investigations to the western part of Northern Cape Province and the adjacent Western Cape Province to seek further possible instances of unrecorded presence of warthogs and potentially infected ticks, or simply to explore the prevalence and distribution of *Ornithodoros* species and virus irrespective of the presence of suids, but this was precluded by COVID-19 travel restrictions. Likewise, it was intended to extend observations to the eastern Free State Province and the adjacent KwaZulu-Natal Province to determine how far northwards the distribution of *O. (P.) zumpti* extends from Eastern Cape Province and how far west and south the distribution of *O. (O.) phacochoerus* extends (Figure 6), but this was also precluded by travel restrictions.

Since the present study was intended merely to seek evidence of the circulation of ASFV in warthogs and ticks beyond the controlled area in South Africa, a definitive analysis of the mechanisms of spread of the sylvatic cycle is not possible. Nevertheless, it is clear that opportunities for the spread of infection include translocation of live warthogs and transportation of carcasses, as well as informal trade in potentially infected pigs and pork products [137,138]. In fact, the genotype IV ASFV isolate with GenBank accession number AF449477 in Figure 4, was obtained from a warthog carcass confiscated at a roadblock in 1999 while being transported without a veterinary permit (L. Heath, unpublished).

Despite the evidence of widespread sylvatic circulation of ASFV beyond the controlled area obtained in the present project and the fact that multiple types of the virus are involved, there was no indication of dispersal and establishment of tick populations beyond their expected species distribution ranges [21]. Thus, a high prevalence of ASFV infection was detected in *O. compactus* within its historic distribution range, but no other tick species were encountered during the admittedly limited sampling of burrows in this area. Even if historically there had been circulation of ASFV between *O. compactus* and the Cape warthog (*Phacochoerus aethiopicus aethiopicus*) that became extinct in 1896 [139], it is unlikely that the virus would have been maintained solely by transovarial transmission in ticks in the absence of viraemic hosts until introductions of the Common warthog commenced more than six decades later [15,52].

Unlike ixodids, larval ticks of the subgenus *Ornithodoros* tend to moult directly into the first nymphal instar without feeding and nymphs undergo 2–8 moults; the number varies with species and may be reduced where early nymphal instars obtain inadequate blood meals. Furthermore, argasid ticks including *Ornithodoros* species do not generally attach to their hosts to feed over a period of days to weeks, but engorge rapidly, within minutes to hours, while their hosts are at rest and then detach to moult to the succeeding instar over a period of weeks to months or to lay eggs as adult females [43,45]. However, ticks that have not completed feeding may be passively conveyed out of burrows when the hosts leave in the mornings, and 46/129 (35.7%) warthogs shot at monthly intervals over the course of a year at three sites in South Africa and Namibia were found to carry a total of 616 *Ornithodoros* nymphs and 2 adults [140,141,142]. It can be surmised that nymphs more readily become enmeshed and cling to the coats of warthogs while the heavier adult ticks have a greater tendency to fall off.

Warthogs are non-migratory and non-territorial, so matriarchal family groups may have overlapping home ranges of approximately 20–170 hectares in different parts of southern Africa. They adapt disused burrows of antbears and certain other animals such as porcupines, or culverts and erosion gullies, for use as shelters at night. They display some tenure of burrows but change occupancy frequently, utilizing up to 10 different shelters [143]. At least 26 other species of mainly small vertebrates have been recorded as utilizing antbear burrows in South Africa [144], so that regular traffic of warthogs and other animals between burrows within relatively short distances of each other could account for localized spread of ticks and virus. However, calculations made for long-distance dispersal of immature *Ornithodoros* ticks parasitic on sea birds indicate that the probability is very small that dispersed ticks would successfully give rise to adult male and female ticks capable of breeding at remote locations [145]. The probability that breeding adult tick vectors infected by a pathogen could successfully be established at a remote site is even smaller; there is a greater chance of a translocated tick transmitting infection than there is of establishing a viable breeding colony of ticks. This model seems to fit the pattern of tick infection observed in the present study. It is planned to conduct vector competence tests on selected tick species and follow-up field studies at provincial level to determine the full extent and mechanisms of spread of sylvatic circulation of ASFV in South Africa.

## Figures and Tables

**Table 1:** Summary of locations where *Ornithodoros* ticks were collected, numbers of ticks collected and pools tested and found positive for African swine fever virus nucleic acid by qPCR, genotypes of virus identified, numbers of tick pools subjected to 16S rRNA sequencing, and species of ticks identified.

		Location		Collection	Ticks	Pools	ASFV qPCR	ASFV p72	16S rRNA	
Location	Province ^1^	Type	QDGC ^2^	Sites	Collected	Tested	Positive	Genotype	Sequences	Tick Species
**Locations inside ASF controlled area**										
LP01	LP	Farm	E029S022CB	1	218	7			6	*O. (O.) waterbergensis*
LP02	LP	Farm	E027S024CA	1	2	1				*O. (O.) waterbergensis*
LP03	LP	Farm	E026S024DB	3	160	27	1	XXII	2	*O. (O.) waterbergensis*
GKNP01	LP	Nature Reserve	E031S024AC	1	82	12	1	XX	12	*O. (O.) phacochoerus*
GKNP02	LP	Nature Reserve	E031S022CA	1	316	46			2	*O. (O.) phacochoerus*
GKNP03	LP	Nature Reserve	E031S022CA	1	26	4			2	*O. (O.) phacochoerus*
GKNP04	LP	Nature Reserve	E031S023CC	1	192	64			2	*O. (O.) phacochoerus*
GKNP05	LP	Nature Reserve	E031S023DC	1	115	15			2	*O. (O.) phacochoerus*
GKNP06	LP	Nature Reserve	E031S023CD	1	60	11			1	*O. (O.) phacochoerus*
GKNP07	MP	Nature Reserve	E031S024BA	1	104	31	2	XXI	31	*O. (O.) phacochoerus*
GKNP08	MP	Nature Reserve	E031S024BA	1	150	26			1	*O. (O.) phacochoerus*
GKNP09	MP	Nature Reserve	E031S024BC	1	58	10			1	*O. (O.) phacochoerus*
GKNP10	MP	Nature Reserve	E031S024BC	1	23	8			1	*O. (O.) phacochoerus*
GKNP11	MP	Nature Reserve	E031S024DB	1	129	36			1	*O. (O.) phacochoerus*
GKNP12	MP	Nature Reserve	E031S024DD	1	84	8			1	*O. (O.) phacochoerus*
GKNP13	MP	Nature Reserve	E031S024DB	1	157	15			1	*O. (O.) phacochoerus*
GKNP14	MP	Nature Reserve	E031S025BA	4	558	25			2	*O. (O.) phacochoerus*
GKNP15	MP	Nature Reserve	E031S025BA	1	56	10			2	*O. (O.) phacochoerus*
GKNP16	MP	Nature Reserve	E031S025BC	1	219	57			1	*O. (O.) phacochoerus*
GKNP17	MP	Nature Reserve	E031S025BD	1	120	14			1	*O. (O.) phacochoerus*
GKNP18	MP	Nature Reserve	E031S025BD	4	254	14	4	XX, XXI	4	*O. (O.) phacochoerus*
NWP01	NWP	Farm	E026S024CD	5	148	25	3	Ia	3	*O. (O.) moubata*
NWP02	NWP	Farm	E026S025AA	3	12	1			1	*O. (O.) moubata*
NWP03	NWP	Farm	E026S025AA	1	136	10	2	Ib	3	*O. (O.) moubata*
**Locations outside ASF controlled area**										
NWP04	NWP	Nature Reserve	E026S025CA	1	7	2	1	Ib	1	*O. (O.) moubata*
NWP05	NWP	Farm	E026S025CA	2	227	37	6	Ib	5	*O. (O.) moubata*
MP01	MP	Nature Reserve	E029S025CA	10	0					
GP01	GP	Nature Reserve	E028S025BC	1	8	5			4	*O. (O.) moubata*
GP02	GP	Nature Reserve	E028S025AD	3	92	6			2	*O. (O.) moubata*
GP03	GP	Farm	E028S025CB	1	1	1			1	*O. (O.) moubata*
GP04	GP	Nature Reserve	E028S025BA	1	10	5			5	*O. (O.) moubata*
GP05	GP	Nature Reserve	E028S025BC	1	10	4			2	*O. (O.) moubata*
GP06-GP07	GP	Farms	E027S026CB	2	0					
GP08-GP11	GP	Farms	E027S026CA	4	0					
GP12-GP13	GP	Farms	E028S025DA	2	0					
GP14-GP18	GP	Farms	E028S025CB	5	0					
MNP01	NCP	Nature Reserve	E024S029AB	6	287	54	4	Ia, Ic	53	*O. (O.) compactus*
NCP01	NCP	Farm	E024S028BC	3	316	51	19	Ia, Ib, Ic ^3^	19	*O. (O.) compactus*
NCP02	NCP	Farm	E024S028AD	2	86	11	3	Ia,b	4	*O. (O.) compactus*
NCP03	NCP	Nature Reserve	E024S028BC	6	65	9			1	*O. (O.) compactus*
NCP04 ^4^	NCP	Nature Reserve	E024S028CB	1	32	3	1	Ia	3	*O. (O.) compactus*
NCP04 ^4^	NCP	Nature Reserve	E024S028CB	1	27	1			1	*O. (O.) kalahariensis*
NCP05	NCP	Farm	E023S028DD	1	53	2			1	*O. (O.) compactus*
NCP06	NCP	Farm	E022S028CC	3	31	3	1	I	1	*O. (O.) compactus*
FSP01	FSP	Nature Reserve	E024S028DD	1	2	1			1	*O. (O.) compactus*
FSP02	FSP	Farm	E025S029AC	3	97	7	1	Ic	3	*O. (O.) compactus*
FSP03	FSP	Farm	E024S029BD	1	8	1	1	III	1	*O. (O.) compactus*
AENP01	ECP	Nature Reserve	E025S033BD	1	7	1			1	*O. (P.) zumpti*
AENP02	ECP	Nature Reserve	E025S033BD	1	234	23			23	*O. (P.) zumpti*
AENP03	ECP	Nature Reserve	E025S033DB	2	91	5			5	*O. (P.) zumpti*
ECP01	ECP	Farm	E024S033AB	2	8	2			1	*O. (O.) noorsveldensis*
				105	5078	711	50		221	

^1^ ECP = Eastern Cape Province; FSP = Free State Province; GP = Gauteng Province; LP = Limpopo Province; MP = Mpumalanga Province; NCP = Northern Cape Province; NWP = North West Province. ^2^ QDGC = Quarter degree grid cell. ^3^ Three tick pools yielded ASFV genotypes Ia plus Ib. ^4^ Two species of tick were collected on same property.

**Table 2:** Abridged evolution of the taxonomy of the Afrotropical members of the subgenus *Ornithodoros (Ornithodoros) (Acari: Ixodida: Argasidae: Ornithodorinae*).

Original Description	Walton, 1962	Van der Merwe, 1968	Walton, 1979	Bakkes et al., 2018 (Type locality)
**Eyes absent: *Ornithodoros moubata* group/complex**				
*Argas moubata* Murray, 1877	*O. moubata*	*O. moubata moubata*	*O. moubata*	*O. moubata* (South Africa)
	*O. porcinus porcinus*	*O. moubata porcinus*	*O. porcinus porcinus*	*O. porcinus* (Tanzania)
				*O. phacochoerus* (South Africa)
				*O. waterbergensis* (South Africa)
	*O. porcinus. domesticus*		*O. porcinus domesticus*	
			*O. porcinus avivora*	
	*O. apertus*	*O. moubata apertus*	*O. apertus*	*O. apertus* (Kenya)
	*O. compactus*	*O. compactus*	*O. compactus*	*O. compactus* (South Africa)
**Eyes present: *Ornithodoros savignyi* group/complex**				
Argas savignyi Audouin, 1827 *	O. savignyi	O. savignyi	*O. savignyi*	*O. savignyi* (Egypt)
*O. pavimentosus*, Neumann, 1901 ^†^				*O. pavimentosus* (South Africa)
				*O. kalahariensis* (South Africa)
				*O. noorsveldensis* (South Africa)

* Subsequently transferred to genus *Ornithodoros,* Koch 1844. ^†^ Synonymized with *O. savignyi,* Theiler and Hoogstraal, 1955; resurrected, Bakkes et al., 2018.

## Data Availability

The original contributions presented in the study are included in the article, further inquiries can be directed to the corresponding author.
